# Emergency Department Transvenous Pacemaker Placement Complicated by Tricuspid Mass

**DOI:** 10.5811/cpcem.53214

**Published:** 2026-05-23

**Authors:** Kevin Molyneux, Nick Krejchi, Matthew Fulton, Mina Youssef

**Affiliations:** * Columbia University, Department of Emergency Medicine, New York, New York; † Texas Tech University Health Sciences Center of El Paso, Department of Emergency Medicine, El Paso, Texas; ‡ Texas Tech University Health Sciences Center of El Paso, Department of Cardiology, El Paso, Texas

**Keywords:** transvenous pacemaker, cardiac myxoma, bradycardia, emergency pacing

## Abstract

**Case Presentation:**

Temporary transvenous pacemaker placement is frequently performed in the emergency department for the management of symptomatic bradyarrhythmias. We report the case of a 93-year-old male who presented with profound bradycardia, hypotension, and altered mental status requiring emergent pacing. Initial transcutaneous pacing achieved hemodynamic improvement but necessitated escalation to transvenous pacing due to patient discomfort and high current requirements. During attempted transvenous pacemaker placement, resistance was encountered and capture could not be achieved despite appropriate technique. Subsequent cardiology consultation and imaging revealed an undiagnosed tricuspid valve myxoma obstructing catheter advancement.

**Discussion:**

This case highlights a rare mechanical complication of transvenous pacemaker placement caused by an intracardiac mass. Awareness of structural cardiac pathology as a potential cause of pacemaker placement failure is critical, particularly when resistance is encountered despite correct procedural technique.

## CASE PRESENTATION

A 93-year-old male with a history of hypertension, pulmonary embolism on apixaban, chronic hypoxic respiratory failure requiring home oxygen, and benign prostatic hyperplasia was brought to the emergency department (ED) by emergency medical services (EMS) for bradycardia, hypotension, and altered mental status. The EMS responders reported an initial blood pressure of ~70/40 millimeters of mercury (mm Hg), which improved to ~90/50 mm Hg after fluid resuscitation. His initial electrocardiogram (ECG) showed junctional bradycardia.

On arrival, the patient was lethargic, and he was immediately placed on transcutaneous pacing, achieving electrical and mechanical capture at a rate of 70 beats per minute with 110 milliamperes (mA). His blood pressure improved to 116/97 mm Hg during pacing, his mental status improved, and his lethargy resolved after the initiation of external pacing. However, due to the high current required for perfusion and the patient’s discomfort, a temporary transvenous pacemaker was indicated.

A 6 French Cordis sheath was placed in the right internal jugular vein using the modified Seldinger technique. Wire placement was confirmed via point-of-care ultrasound, and blood was easily aspirated. An electrode catheter was then introduced through the Cordis.

At approximately 22 cm, resistance was encountered, and capture did not improve on the monitor despite an output of 20 mA. The electrode catheter was removed, and a second attempt was made. Once again, blood was aspirated without resistance, but the second electrode catheter placement was also unsuccessful. Interventional cardiology was consulted and, after multiple attempts, successfully placed a transvenous pacemaker.

**Image 1:**
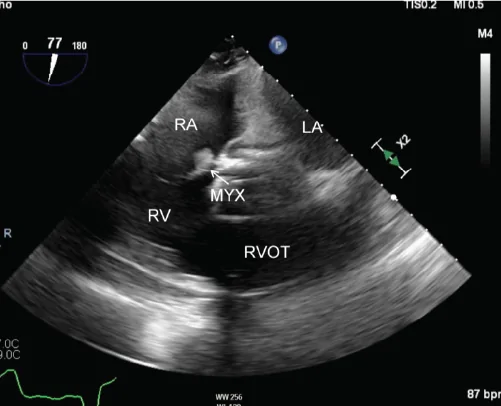
Transesophageal echocardiogram, mid-esophageal right ventricular inflow-outflow tract view. *RA*, right atrium; *RV*, right ventricle; *LA*, left atrium; *RVOT*, right ventricular outflow tract; *MYX*, myxoma.

**Image 2:**
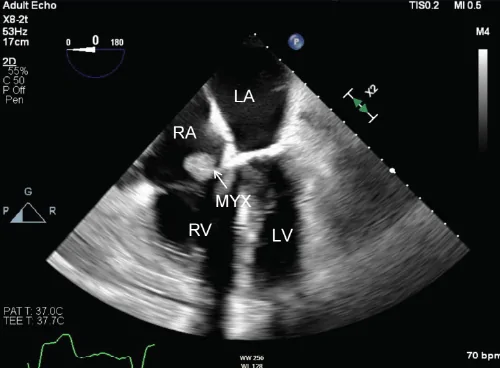
Transesophageal echocardiogram, mid-esophageal-chamber view. *RA*, right atrium; *RV*, right ventricle; *LA*, left atrium; *MYX*, myxoma.

During the patient’s inpatient stay, a transesophageal echocardiogram revealed a 1.8 cm x 1.6 cm mobile globular mass attached to the atrial side of the septal tricuspid valve leaflet by a short stalk, consistent with a myxoma (Images 1 and 2). Mild tricuspid regurgitation was also noted.

The patient was pacemaker-dependent, developing asystole within five seconds of pacing cessation. A permanent dual-chamber pacemaker was subsequently implanted with resolution of bradycardia. At one- and two-month outpatient follow-up, the patient remained asymptomatic.

## DISCUSSION

Temporary transvenous pacemaker placement is a critical emergency procedure for managing brady-arrhythmias. This case highlights a unique complication where a right atrial intracardiac mass interfered with standard transvenous pacemaker placement. Resistance during electrode catheter advancement and failure to achieve capture necessitated procedural intervention by cardiology, ultimately leading to the discovery of a tricuspid valve mass via transesophageal echocardiogram.

Identifying the mass in the ED proved challenging due to the required use of multifunction pads blocking commonly obtained windows to continue pacing the patient and prevent critical clinical instability. Myxomas, although rare, are the most common primary cardiac tumors; they can significantly obstruct blood flow and, therefore, can theoretically obstruct catheter or wire advancement and/or alter their trajectory.^1^–^3^ Right atrial myxomas are even rarer, accounting for 15–20% of all cardiac myxomas.^3^ In this case, the mass on the tricuspid valve likely created a physical barrier, complicating wire placement and necessitating cardiology consultation for successful permanent pacemaker insertion.

*CPC-EM Capsule*

What do we already know about this clinical entity?

*Temporary transvenous pacing is routinely used in the emergency department for unstable bradyarrhythmias when transcutaneous pacing is ineffective.*

What makes this presentation of disease reportable?

*An occult tricuspid valve myxoma caused unexpected resistance and failure of transvenous pacemaker placement.*

What is the major impact of the image(s)?

*Failure or resistance during transvenous pacing should raise concern for intracardiac structural pathology.*

How might this improve emergency medicine practice?

*Early recognition of rare mechanical causes of pacing failure will ensure timely cardiology consultation.*
